# Enhancing EEG-Based MI-BCIs with Class-Specific and Subject-Specific Features Detected by Neural Manifold Analysis

**DOI:** 10.3390/s24186110

**Published:** 2024-09-21

**Authors:** Mirco Frosolone, Roberto Prevete, Lorenzo Ognibeni, Salvatore Giugliano, Andrea Apicella, Giovanni Pezzulo, Francesco Donnarumma

**Affiliations:** 1 Institute of Cognitive Sciences and Technologies, National Research Council, Via Gian Domenico Romagnosi, 00196 Rome, Italy; mirco.frosolone@istc.cnr.it (M.F.); lorenzo.ognibeni@istc.cnr.it (L.O.);; 2 Department of Electrical Engineering and Information Technology (DIETI), University of Naples Federico II, 80125 Naples, Italy; roberto.prevete@unina.it (R.P.); salvatore.giugliano2@unina.it (S.G.); andrea.apicella@unina.it (A.A.); 3 Department of Computer, Control and Management Engineering ‘Antonio Ruberti’ (DIAG), Sapienza University of Rome, 00185 Rome, Italy

**Keywords:** neural manifold analysis, motor imagery BCI, EEG-based BCI, transfer learning

## Abstract

This paper presents an innovative approach leveraging Neuronal Manifold Analysis of EEG data to identify specific time intervals for feature extraction, effectively capturing both class-specific and subject-specific characteristics. Different pipelines were constructed and employed to extract distinctive features within these intervals, specifically for motor imagery (MI) tasks. The methodology was validated using the Graz Competition IV datasets 2A (four-class) and 2B (two-class) motor imagery classification, demonstrating an improvement in classification accuracy that surpasses state-of-the-art algorithms designed for MI tasks. A multi-dimensional feature space, constructed using NMA, was built to detect intervals that capture these critical characteristics, which led to significantly enhanced classification accuracy, especially for individuals with initially poor classification performance. These findings highlight the robustness of this method and its potential to improve classification performance in EEG-based MI-BCI systems.

## 1. Introduction

The field of Brain–Computer Interfaces (BCIs) has witnessed significant advancements over the past decades, forecasting exceptional achievements in bioengineering applications [1,2,3]. BCIs rely on the measurement of neural activity as a method to determine the user’s intentions. Suitable command sequences can be produced using BCI applications, including those based on non-invasive methods such as functional magnetic resonance imaging (fMRI), magnetoencephalography (MEG), and electroencephalography (EEG). Among these, the most widely used BCI systems leverage EEG signals due to their simplicity, affordability, and ability to support effective real-time implementations thanks to their intrinsic high temporal resolution [4,5]. BCIs are particularly effective in motor imagery (MI) tasks, where users imagine performing motor movements to control external devices. Typically, in an MI-based BCI, the user is required to imagine the motor action related to their body parts (e.g., left hand, right hand, feet, or tongue). It is widely accepted that the mental imagination of movements involves brain regions similar to those engaged in the actual execution of these movements [6,7]. Differently from the physical execution, in MI the movement is blocked at a corticospinal level. However, functional brain imaging studies demonstrated that patterns of brain activity during motor imagery are similar to those observed during actual movement execution. Specifically, an activation is observed in various structures involved in the early stages of motor control, such as motor *programming* and *planning*. For example, imagining right-hand or left-hand movements activates the contralateral hand area, the top-central for feet, and the parietofrontal for tongue MI [8,9,10,11]. This neural activation can be readily detected through the EEG signals [12]. MI-BCI systems prominently depend on sensorimotor rhythms (SMR), event-related potentials (ERPs), visually evoked potentials (VEPs), and slow cortical potentials (SCPs). Among these, SMR-based BCI systems offer significant freedom in real-time control and motor imagery activities, such as movements of the tongue, hand, arm, and feet [13]. Decoding the recorded EEG signals and mapping the corresponding MI to a command for an external device is the primary challenge in EEG-based MI-BCI systems. The EEG-based MI-BCIs are crucial for designing systems that enable specific activities such as controlling and governing wheelchairs [14], home appliances, speech synthesizers, robotic prostheses [15], post-stroke rehabilitation [16,17], digital computers, and competitive or collaborative games [18,19,20,21].

An EEG-based MI-BCI system encompasses a pipeline involving various steps [18]: (a) Signal acquisition: EEG signals are collected from the scalp using specialized hardware while the user performs MI tasks. (b) Signal processing: the goal is to increase the signal-to-noise ratio of the weak EEG signals, which are often contaminated by artifacts and interferences such as muscle movements, eye blinks, heartbeats, and powerline noise. (c) Feature extraction and selection: this involves extracting relevant properties in the time domain, frequency domain, or time–frequency domain and selecting those that are most successful in representing the task. (d) Classification: the extracted features are used to decode the EEG signals. (e) Control: proper commands are then sent to an external device, such as a wheelchair, based on the decoded EEG signals.

In this work, the focus is on the crucial step in the BCI pipeline: feature extraction. Extracted suitable features correspond to vital information encapsulated in the signal. Various techniques can be employed to extract the most informative parts of the input signals, including fast Fourier transform (FFT) [22], autoregressive model (AR) [23], Common Spatial Pattern (CSP) [24], and Wavelet Transform (WT) [25]. This step corresponds to a *Neural Manifold Analysis* (NMA) [26], where EEG signals are denoised and reorganized, reducing the high-dimensional input signal space to a more manageable lower-dimensional space [27]. The classification process then converts the features encoded in the manifold generated by the feature extractor into commands. The more readable this manifold is, the easier the classification becomes. BCI classification techniques translate these discriminatory features into decoded motor activities such as tongue movement, left–right movement, and foot movement. Several classification methods like artificial neural networks (ANN), linear discriminant analysis (LDA), k-nearest neighbors (k-NN), support vector machines (SVM), Gaussian Naive Bayes (GNB), and deep learning (DL) have been used for MI-BCI systems [28]. A major challenge in BCIs is that different users have varying neuronal responses to the same stimulus, and even the same user can exhibit different neuronal responses to the same stimulus at different times or conditions. Additionally, calibrating the BCI system requires acquiring a large number of subject-specific labeled training examples for each new subject, which is both time-consuming and expensive.

To address these issues, different approaches involving Transfer Learning have been explored [29,30,31,32]. Those studies use data extracted from one or more source domains to help construct the representation manifold in the target domain, effectively addressing these problems. In [33], the authors employ DL models for feature extraction in EEG signals, achieving notable improvements in classification performance through the use of convolutional neural networks. In [34], a DL approach is used to select samples in the source neuronal domain that are closest to the data in the target manifold domain, assigning them high correlation weights. In [35], the authors adapt a DL approach to utilize pre-trained features from different datasets. Additionally, in [36], a method to extract cross-channel specific-mutual features is proposed, further enhancing cross-subject generalization.

Nevertheless, several recent studies have explored various methods to improve EEG-based BCI systems, focusing on feature extraction and classification while highlighting the importance of addressing cross-subject variability [4,37]. For instance, in [38], the authors proposed a method for regularizing CSP features to enhance the robustness of BCI systems against subject-specific variations. This is crucial since EEG signals often exhibit large inter-subject variability, making consistent classification challenging. Importantly, studies utilized NMA techniques, such as Principal Component Analysis (PCA), to detect spatio-temporal features in EEG inputs, which are useful for increasing task classification accuracy [39,40]. Additionally, works like [41] focused on time–frequency feature extraction for MI tasks, emphasizing the importance of selecting optimal time intervals to boost classification accuracy. Furthermore, ref. [42] introduced a subject-independent approach for MI classification, demonstrating the potential of using data from multiple subjects to improve overall classification accuracy. CSP is a widely used technique for spatial filtering in EEG signals, aiming to maximize the variance difference between two classes. Despite its success, CSP’s reliance on data from the same subject and specific time intervals often limits its generalizability across different subjects or sessions.

Consequently, feature extraction in motor imagery has evolved from single-domain (time, frequency, or spatial) approaches to multi-domain fusion, especially combining spatial and frequency domain information. Thus, methods for NMA, are employed to determine patterns of covariance in participants’ responses, extracting separate features for each class and subject.

In this study, it is proposed a novel approach leveraging NMA to identify optimal time intervals for feature extraction, which are critical for improving classification performance [37]. NMA involves analyzing the EEG signal in a multi-dimensional feature space to detect intervals that capture class-specific and subject-specific characteristics. By applying state-of-the-art feature extraction algorithms within these identified ranges, the goal is to improve the discriminative power of the extracted features. Furthermore, this work addresses the challenge of subjects with poor classification performance by cross-validating the extracted features across different subjects. By incorporating features from subjects with high classification accuracy, significant improvements are achieved for subjects that initially exhibit poor performance. Traditionally, NMA is used to derive a reduced manifold, enabling more effective feature extraction and, consequently, better classification. In the present paper, however, an innovative use of NMA is proposed: using a separability measure on the neural manifolds, it is possible to identify specific temporal segments of the EEG signal, where, based on manifold analysis, we can extract more relevant features. This method allows for the construction of multiple manifolds over specific temporal segments, which can then be combined into a more complex overall manifold. The resulting manifold demonstrates enhanced discriminability compared with traditional approaches, making it more effective for classification tasks. To demonstrate the effectiveness of the proposed approach, particularly suited for the classification of oscillatory components of SMR during MI tasks, results from two datasets are presented [43,44,45]: Graz Dataset 2b, which highlights the robustness of the NMA pipelines in binary classification, and Graz Dataset 2a, a key benchmark for motor imagery, including a more complex four-class classification problem. This work develops NMA processing pipelines by building on the insights gained from the winners of BCI Competition IV [46,47], while also integrating recent advances in deep learning methods [33]. The performance results in both two-class and four-class motor imagery tasks in the selected datasets highlight the significant potential of NMA to improve cross-class and cross-subject classification.

The present paper is organized as follows: Section 2, Materials and Methods, provides a detailed explanation of the MNA pipelines used in the experiments. This is followed by the results section (Section 3), which is divided into two parts: Section 3.1 presents results from Graz Dataset 2b, while Section 3.2 focuses on Graz Dataset 2a. Finally, Section 4 summarizes the findings achieved with the proposed method and discusses potential avenues for future research and development.

## 2. Materials and Methods

Our starting point is the FBCSP Algorithm, which was the winner of the Graz BCI Competition [44,45] and has since become a gold standard in the BCI community. As previously mentioned, the core components of an EEG BCI pipeline include several critical steps. In this paper, the following FBCSP pipeline is employed:

- Signal processing: Filter Bank.
- Feature extraction: common spatial pattern algorithm.
- Feature selection: mutual information-based best individual feature.
- Classification: quadratic discriminant analysis classifier.

**Figure 1:**
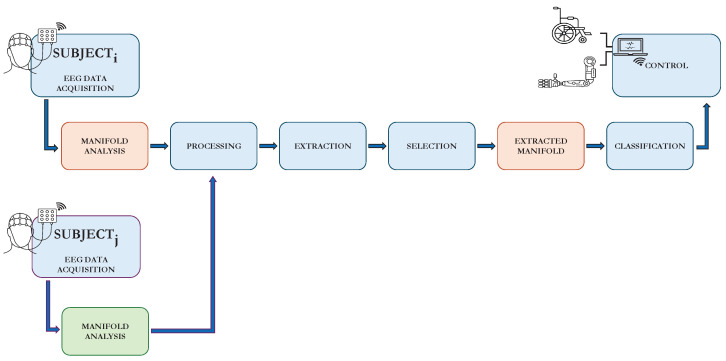
Depiction of a BCI system, including modules for Signal Acquisition, Feature Extraction, Feature Selection, and Classification. The system’s ability to discriminate between classes enables it to send commands to a control system, such as a wheelchair, prosthetic hand, or other BCI-controlled devices. In the presented approach, this process is augmented with NMA performed on the EEG signal to design better features that enhance the classification system. Furthermore, this information, especially for poorly performing subjects, can be improved by incorporating analysis from other subjects.

In Figure 1, a depiction of the process flow of a BCI system, including its key modules, is presented. Additionally, in this paper, the standard pipeline was enhanced with further NMA modules, leading to notable improvements in classification accuracy. In the following subsections, the modules of the system used to test the methodology will be described.

### 2.1. Signal Processing: Filter Bank

After acquisition, the input EEG signal can be represented as a time series $X\in R^{N_{ch}\times T}$, where $N_{ch}$ is the number of channels and *T* is the number of time samples acquired. The signal undergoes the first of a series of preprocessing steps. A filter bank comprising multiple (B=9) Chebyshev Type II band-pass filters is utilized, i.e., nine Chebyshev Type II filters, each one designed for a specific band, as described in [45]. Each filter has a 4 Hz-wide pass band, resulting in nine non-overlapping bands from 4 Hz to 40 Hz, denoted as ${\left\{\left(4b,4b+4\right)\right\}}_{b=1}^{B}$. The best bands are selected on a subject-by-subject basis during feature selection. The attenuation in the stop band is set to −20 dB, and the filter order is 4, achieving a sharp roll-off in the frequency response.

Thus, starting with an input signal X, the result of the filtering is an output $\chi \in R^{N_{ch}\times T\times B}$. The chosen band-pass frequency ranges allow for a stable frequency response and coverage of the 4–40 Hz range.

### 2.2. Feature Extraction: Common Spatial Pattern Algorithm

The CSP algorithm creates a reduced space to maximize the separability of labeled samples. Typically, CSP approaches are applied when two classes are present, but multi-class extensions are possible [48]. In this work, the one-versus-rest (OVR) approach is adopted [45], which allows for discrimination among an arbitrary number *K* of classes. In the OVR–CSP method, selecting *m* CSP components enables the construction of projection matrices $W_{k}^{b}\in R^{N_{ch}\times 2m}$, one for each band *b* and for each class *k*, which can then be applied to the filtered data χ. These projection matrices are derived from a sample set of labeled data (training set) during the training phase. Consequently, each filter band and class has an associated projection matrix $W_{k}^{b}$. In the transformed space, the first *m* CSP components have maximum variance associated with class *k* and minimum variance for the remaining classes, while the last *m* components have minimum variance for class *k* and maximum variance for others; see [45,48] for details.

The *N* samples in the input set can be partitioned into *K* sets ${\left\{\Pi_{k}\right\}}_{k=1}^{K}$, where $\Pi_{k}$ denotes the sample set of the *k*-th class containing $N_{k}$ data points. For each band, selecting the samples $\chi_{n}$ belonging to class *k*, the class covariance matrix can be computed as

(1) $$S_{k}=\frac{1}{N_{k}}\sum_{\chi_{n}\in \Pi_{k}}\chi_{n}\chi_{n}^{T}$$

and the corresponding composite covariance matrix is $S=\sum_{k}S_{k}$. The complete projection matrix $W_{k}$ is obtained by solving the eigenvalue decomposition problem:

(2) $$S_{k}W_{k}=SW_{k}\Lambda_{k}$$

where $\Lambda_{k}$ is a diagonal matrix of the eigenvalues, sorted in ascending order, and $W_{k}$ consists of the corresponding eigenvectors. The final projection matrix $W_{k}^{b}$ is obtained by selecting the first *m* and the last *m* columns of $W_{k}$ (in the reported tests m=2, see [45]).

Each time series $\chi_{n}\in R^{N_{ch}\times T}$ for a single trial and band is projected into a new space using $W_{k}^{b}$, resulting in $V={\left(W_{k}^{b}\right)}^{T}\chi_{n}\in R^{2m\times T}$. From the projected trial *V*, a covariance matrix $A=VV^{T}$ is computed. From this matrix *A*, a vector is obtained by selecting the elements of the diagonal (the variances) $a=A_{1}^{1},A_{2}^{2},\cdots ,A_{2m}^{2m}$. Renormalizing this vector and tacking the logarithmic values ${\tilde{a}}_{k}=log\left(a/∥a∥\right)$, a feature vector $a_{k}$ of 2m elements for each class *k* is obtained. Concatenating features from each class results in an element of the feature space:

(3) $${\tilde{z}}^{b}=\left({\tilde{a}}_{1},\cdots ,{\tilde{a}}_{k}\right)$$

with ${\tilde{z}}^{b}\in R^{m\times 2K}$, i.e., a CSP feature with 2K values. Notice that these features are related to one band *b*, thus the total features are $\tilde{z}=\left({\tilde{z}}_{1},\cdots ,{\tilde{z}}_{B}\right)$, organized in a space $\tilde{Z}\in R^{2mB\times K}$. These F=2mB features are subjected to the feature selection phase, described in the next subsection.

### 2.3. Feature Selection: Mutual Information-Based Best Individual Feature

The feature selection algorithm is crucial for identifying discriminative features in $\tilde{Z}$ for the subject’s task. The mutual information-based best individual feature (MIBIF) was the winning algorithm of the BCI Competition [45,49]. Feature selection is executed on the training set by selecting the most discriminative CSP features based on the mutual information computed between each feature and the corresponding motor imagery classes. A parameter is chosen to select a number of *D* features. The *N* samples in the manifold can be partitioned into *K* sets ${\left\{\Pi_{k}\right\}}_{k=1}^{K}$, where $\Pi_{k}$ denotes the sample set of the *k*-th class containing $N_{k}$ data points.

A set of F=2m·B·K features, ${\tilde{z}}_{n}=\left({\tilde{z}}_{n}^{1},\cdots ,{\tilde{z}}_{n}^{F}\right)\in R^{F}$, is associated with an input trial $x_{n}$ belonging to a specific class *k*. For each j∈[1,⋯F], the mutual information $M\left({\tilde{z}}_{n}^{j};k\right)$ with each class label *k* can be computed. It is possible to define $M\left({\tilde{z}}_{n}^{j};k\right)=H\left(k\right)-H\left(k|{\tilde{z}}_{n}^{j}\right)$, where $H\left(k\right)=-\sum_{k=1}^{K}P\left(k\right)\log_{2}P\left(k\right)$ is the entropy over the choice of *k*, and the conditional entropy $H\left(k|{\tilde{z}}_{n}^{j}\right)=-\sum_{k=1}^{K}p\left(k|{\tilde{z}}_{n}^{j}\right)\log_{2}p\left(k|{\tilde{z}}_{n}^{j}\right)$.

The conditional probability $p\left(k|{\tilde{z}}_{n}^{j}\right)$ of class *k* given the *j*-th feature ${\tilde{z}}_{n}^{j}$ is estimated. Initially, the probability is constructed such that, given a class *k*, the *j*-th feature ${\tilde{z}}_{n}^{j}$ is found:

(4) $$p\left({\tilde{z}}_{n}^{j}|k\right)=\frac{1}{N_{k}}\sum_{{\tilde{z}}_{k}\in \Pi_{k}}\phi \left({\tilde{z}}_{n}^{j}-{\tilde{z}}_{k}^{j},h\right)$$

where $\phi \left(x,h\right)=\frac{1}{\sqrt{2\pi }}e^{-\left(x^{2}/2h^{2}\right)}$ is a Gaussian kernel with an attenuation parameter *h*. Then the probability is computed using Bayes’ theorem, $p\left(k|{\tilde{z}}_{n}^{j}\right)\propto p\left({\tilde{z}}_{n}^{j}|k\right)p\left(k\right)$, where $p\left(k\right)=N_{k}/N$. The resulting selected features z lie in the manifold $Z\in R^{D}$. In the current implementation, D=4·K. To keep a conservative approach, the twin CSP feature is preserved if it is not included in this set. Thus, in the case where none of the twin CSP features were already included in the set *D* could raise to D=8·K (see [49]).

### 2.4. Classification: Quadratic Discriminant Analysis

Quadratic discriminant analysis (QDA) is a widely used approach for classification [50]. Given a manifold $Z\in R^{D}$ with *N* data samples, each data sample $z_{n}\in Z$ belongs to one of *K* classes and is represented by a one-hot encoded label vector $d_{n}$ such that if $z_{n}$ belongs to the *k*-th class, then $d_{n}\left(k\right)=1$, where $d_{n}\in {\left\{0,1\right\}}^{K}$. Once again, the *N* samples in the manifold can be partitioned into *K* sets ${\left\{\Pi_{k}\right\}}_{k=1}^{K}$, where $\Pi_{k}$ denotes the sample set of the *k*-th class containing $N_{k}$ data points. QDA models each class with a multivariate Gaussian distribution:

(5) $$P\left(z_{n}|d_{n}\left(k\right)=1\right)=N_{k}\left(z_{n};\mu_{k},\Sigma_{k}\right)$$

where $\mu_{k}$ and $\Sigma_{k}$ are the mean vector and covariance matrix for each class, respectively. The decision boundaries in QDA are designed to enhance class separability by maximizing the within-class scatter:

(6) $$D_{within}=\sum_{k=1}^{K}\sum_{z_{n}\in \Pi_{k}}\left(z_{n}-\mu_{k}\right){\left(z_{n}-\mu_{k}\right)}^{⊤}$$

and the between-class scatter:

(7) $$D_{between}=\sum_{k=1}^{K}N_{k}\left(\mu_{k}-\mu \right){\left(\mu_{k}-\mu \right)}^{⊤}$$

where μ is the global mean of the *N* input samples. The quadratic discriminant functions can be written as

(8) $$\log P\left(d(k)=1|z\right)\propto \delta_{k}\left(z\right)=-\frac{1}{2}\log\left|\Sigma_{k}\right|-\frac{1}{2}{\left(z-\mu_{k}\right)}^{⊤}\Sigma_{k}^{-1}\left(z-\mu_{k}\right)+\log\left(\frac{N_{k}}{N}\right)$$

from the relation $P\left(d(k)=1|z\right)\propto P\left(z|d(k)=1\right)\cdot P\left(k\right)$. This allows computation of the score of a new signal time series z in the manifold for belonging to the *k*-th class.

### 2.5. Neural Manifold Analysis

In the previous subsections, a procedure for each EEG trial $x\in R^{T}$, considered a time series, was formalized to extract points in a reduced manifold z∈Z in such a way that MI classes can be handled with a multi-class classifier.

In this subsection, an approach utilizing a NMA on EEG data is presented (see Figure 1) to reorganize time series by identifying specific time intervals, capturing class-specific and subject-specific characteristics, and improving class discriminability. This method involves analyzing the EEG signal in a multi-dimensional feature space to detect intervals that best capture the relevant features corresponding to the different MI tasks. Additionally, a cross-validation of the extracted time features across subjects is performed, significantly improving classification accuracy for challenging subjects. This underscores the reliability and potential of the presented method for enhancing cross-subject classification in EEG-based BCI systems.

Different approaches to analyzing acquired neuronal signals involve PCA, PPCA, GPFA [51], demixed PCA [52], pi-Variational Auto Envoders (VAEs) [53], UMAP [54], or frameworks like MIND [55], LFADS [56], and CEBRA [57,58]. NMA aims to uncover the underlying structure of high-dimensional neuronal data by projecting it into a lower-dimensional space where the data’s intrinsic properties are more apparent. Techniques such as PCA [26,40] are applied to reduce the dimensionality of the EEG data while preserving its most significant features. This step transforms the high-dimensional EEG signals into a more manageable lower-dimensional space, capturing the essential patterns and structures inherent in the data.

Movement planning functions in the brain are hypothesized to occur in a low-dimensional subspace of movements called movement primitives, often corresponding to a reduced neuronal manifold. These neuronal primitives enable the control of multiple degrees of freedom of movement with fewer control signals [59,60]. To quantitatively compare the differences between the encoded variables (such as direction and task), is considered an *H*-dimensional neuronal manifold, formed by *H* sub-manifolds identified by a set of elements $\left\{s_{1},\cdots ,s_{H}\right\}$, resulting in a neural set of sub-manifolds or *dictionary* [60,61,62], emerging from the space reduction with PCA. Each EEG trial, i.e., neural trajectories, can be approximated as

(9) $$x\left(t\right)=\sum_{h=1}^{H}c^{h}\left(t\right)\cdot s_{h}$$

with t∈T, where $c^{h}\left(t\right)$ are the coefficients of the decomposition with respect to the sub-manifold *h*. This can be viewed as an *H*-dimensional trajectory in $R^{H}$. For each trajectory, is computed a separability measure in each direction of $R^{H}$ in a supervised manner with respect to the classes of the dataset. The *N* samples in the input set can be partitioned into *K* sets ${\left\{\Pi_{k}\right\}}_{k=1}^{K}$, where $\Pi_{k}$ denotes the sample set of the *k*-th class containing $N_{k}$ data points. For each class, can be selected the coefficient $c_{k}^{h}\left(t\right)$ relative to the trials of the corresponding class $x\in \Pi_{k}$.

Given these trajectories in each sub-manifold, a *separability* measure reflecting the probability of the trajectories being separated is computed, and it is used as a probability of separation among classes. For simplicity, a one-way ANOVA is performed at each time step among the values $c_{k}\left(t\right)$, obtaining a *p*-value that measures the separability of the classes over time. The more this *p*-value approaches zero, the more separable the classes are. Additionally, using a post hoc Tukey test [63], the separability measure $p_{ij}$ between two distinct classes can also be obtained. By studying the trend of these measures, the minimum values where the classes are most separated can be identified. Corresponding to these values, the time *s* of maximum separability among classes ($\arg\min_{t\in T}p\left(t\right)$) and the time $s_{ij}$ of maximum separability between class *i* and class *j* ($\arg\min_{t\in T\;\mathrm{and}\;p(t)<0.05}p_{ij}\left(t\right)$) can be determined.

Whenever *s* and $s_{ij}$ are computed, these times are used to organize new trials x∈{x∣[s−Δt,s+Δt]} or $x\in \{x|\left[s_{ij}-\Delta t,s_{ij}+\Delta t\right]\}$, respectively. On these new trials, the FBCSP procedure is performed to obtain better features and improve classification [64]. Seven selected viable pipelines for trial pre-processing are presented, followed by an explanation of how these pipelines are applied to generate new trials. In the results section, the effectiveness of these pipelines in improving classification accuracy is demonstrated.

### 2.6. NMA Pipelines

In the FBCSP approach [44], the analysis interval corresponds to the entire period considered informative for the task (e.g., in the presented experiments, *T* corresponds to [0.5,2.5], see Section 3). However, using CSP to extract a unique multi-dimensional point over this entire interval may discard valuable information within the noise. Moreover, splitting intervals and aggregating them afterwards can be computationally expensive due to the numerous potential choices, and arbitrary splits may introduce additional problems, discarding critical information. In this framework, a guided procedure that extracts crucial information, enhancing classification, is proposed.

The starting point is to use the entire interval of interest, as done in standard FBCSP. Here, various pipelines are explored to form new trials x in different intervals, aiming to improve the procedure. Once defined *T* as the original motor imagery interval, NMA is performed over all *T* to identify interval of interests for the pipelines: $T_{s}=\left[s-\Delta T,s+\Delta T\right]$, where *s* is the time of maximum separability found with the NMA procedure; $T_{s_{ij}}=\left[s_{ij}-\Delta T,s_{ij}+\Delta T\right]$, where $s_{ij}$ is the maximum time of separability between classes *i* and *j* identified by the NMA procedure, where *i* and *j* are chosen looking at the confusion matrix of a classification with FBCSP procedure; $T_{s_{ij}}^{0}=\left[s_{ij}-\Delta T,s_{ij}+\Delta T\right]$, where $s_{ij}$ is the maximum time of separability between classes *i* and *j* identified by the NMA procedure, where *i* and *j* are chosen looking at the confusion matrix of a classification with Pipeline 0 procedure (see below).

Thus, the following pipelines are realized in this study with NMA:

**FBCSP**:  The entire EEG signal interval *T*, during which the motor imagery task is performed, resulting in trials $x\in R^{N_{ch}\times T}$.
**Pipeline 0**: Reduced EEG trials $x\in R^{N_{ch}\times T_{0}}$ centered on the maximum separability time point among classes.
**Pipeline 1**: For each trial, two time series are obtained: one corresponding to standard FBCSP $x_{1}\in R^{N_{ch}\times T}$ and the other to Pipeline 0 $x_{2}\in R^{N_{ch}\times T_{s}}$. The features obtained from each FBCSP procedure are concatenated and sent to the classifier.
**Pipeline 2**: For each trial, two time series are obtained: $x_{1}\in R^{N_{ch}\times T}$ and $x_{2}\in R^{N_{ch}\times T_{s}}$. These signals are concatenated to form a new time series $x=\left[x_{1},x_{2}\right]\in R^{N_{ch}\times T+T_{s}}$, on which the feature extraction procedure is applied.
**Pipeline 3**: For each trial, two time series are obtained: one corresponding to standard FBCSP $x_{1}\in R^{N_{ch}\times T}$ and the other to $x_{2}\in R^{N_{ch}\times T_{s_{ij}}}$, corresponding to the maximum separability time point between classes *i* and *j*. The features obtained from each FBCSP procedure are concatenated and sent to the classifier.
**Pipeline 4**: For each trial, two time series $x_{1}\in R^{N_{ch}\times T}$ and $x_{2}\in R^{N_{ch}\times T_{s_{ij}}}$ are obtained. These signals are concatenated to form a new time series $x=\left[x_{1},x_{2}\right]\in R^{N_{ch}\times T+T_{s_{ij}}}$, on which the feature extraction procedure is applied.
**Pipeline 5**: For each trial, two time series are obtained: one corresponding to standard FBCSP $x_{1}\in R^{N_{ch}\times T}$ and the other to $x_{2}\in R^{N_{ch}\times T_{s_{ij}}^{0}}$, corresponding to the maximum separability time point between classes *i* and *j*. The features obtained from each FBCSP procedure are concatenated and sent to the classifier.
**Pipeline 6**: For each trial, two time series $x_{1}\in R^{N_{ch}\times T}$ and $x_{2}\in R^{N_{ch}\times T_{s_{ij}}^{0}}$ are obtained. These signals are concatenated to form a new time series $x=\left[x_{1},x_{2}\right]\in R^{N_{ch}\times T+T_{s_{ij}}}$, on which the feature extraction procedure is applied.

Note that the intervals *T*, $T_{s}$, $T_{s_{ij}}$, and $T_{s_{ij}}^{0}$ are all subject-specific. However, combining this information from different subjects can further enhance the accuracy of a BCI system, as demonstrated in Section 3.

## 3. Experimental Results

This section is divided into two subsections, each corresponding to a different dataset. The first subsection covers tests on a two-class dataset, Graz Dataset 2b, while the second subsection focuses on a four-class dataset, Graz Dataset 2a. Results from both datasets are presented and thoroughly discussed.

### 3.1. Tests on Graz Dataset 2b

This part provides a brief description of the Graz Dataset 2b, followed by a report and discussion on the improvements introduced by NMA. Comparisons are made with the approaches that demonstrated the best overall performance across all subjects among the algorithms submitted to the BCI Competition IV [46,47].

#### 3.1.1. Graz Dataset 2b Description

**Figure 2:**
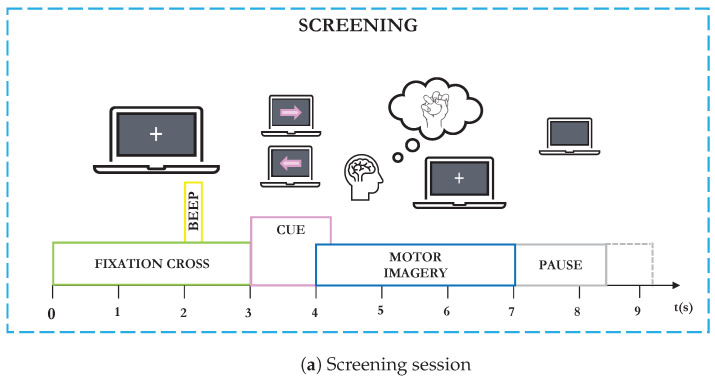

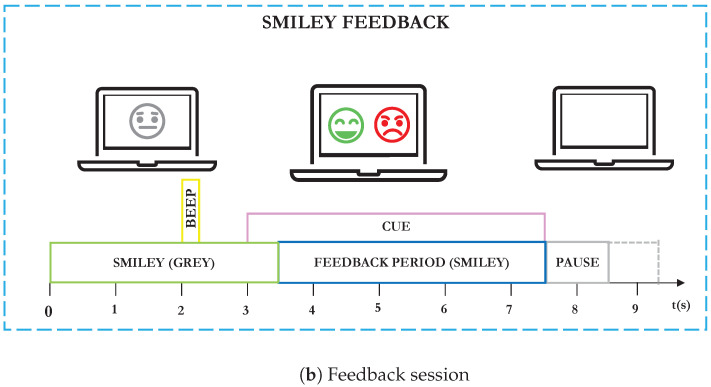
Graz Dataset 2b MI timing scheme of the paradigm. (**a**) Screening session: Subjects sat in front of a computer screen. After t=3 s, a cue in the form of an arrow (left or right) appeared for t=1.25 s, indicating the MI task to perform. Subjects were instructed to continue the MI task until the fixation cross disappeared at t=7 s, followed by a short break with a black screen. (**b**) Feedback session: At the beginning (t=0 s), a gray smiley appeared on the screen. From t=3 s to t=7.5 s, a visual cue was presented, and the subject started the MI hand movement. During this feedback period, the smiley turned green when the motor imagery moved in the correct direction and red if incorrect. After this period, the screen went blank, and a new trial began.

The Graz Dataset 2b [65] consists of EEG recordings from nine right-handed subjects who participated in a two-class motor imagery study: left hand (class 1) and right hand (class 2). EEG data were recorded using three bipolar electrodes placed at positions C3, Cz, and C4. The experiment consisted of five sessions, with the first two dedicated to training without feedback (screening sessions) and the last three incorporating real-time feedback. During the screening sessions, subjects sat in front of a computer screen. After 3 s, a cue in the form of an arrow (pointing left or right) indicating the motor imagery task to perform appeared for 1.25 s. Subjects were instructed to continue the motor imagery task (which involved imagining the movement of either their right or left hand) until the fixation cross disappeared at 7 s. A short pause followed, with a black screen (see Figure 2a). During the feedback session, at the beginning of each trial (second 0), the feedback (a gray smiley) was centered on the screen. At second 2, a short warning beep (1 kHz, 70 ms) was given. From second 3 to 7.5, a visual cue was presented, and depending on the cue, the subjects were required to move the smiley towards the left or right side by imagining the corresponding hand movement. During this feedback period, the smiley turned green when moved in the correct direction and red if incorrect. The smiley’s distance from the origin was adjusted based on the integrated classification output over the past two seconds. Additionally, the classifier output influenced the curvature of the smiley’s mouth, making it appear happy (corners of the mouth upward) or sad (corners downward). At second 7.5, the screen went blank, followed by a random interval between 1.0 and 2.0 s. Subjects were instructed to keep the smiley on the correct side for as long as possible, continuing the MI task throughout the trial (see Figure 2b). The training data comprised the first two sessions (screening) and the third session (with feedback), totaling 240 trials without visual feedback and 160 trials with feedback (named BT). The evaluation data were drawn from the remaining two sessions, consisting of 320 trials (named BE). The dataset is open and freely available at www.bbci.de/competition/iv/ (accessed on 2 February 2024).

#### 3.1.2. Performance Comparison on Graz Dataset 2b

This section illustrates how the MNA approach can be effectively applied in a binary classification task with a limited number of channels (3), showing its potential for improving performance. Table 1 presents the results of 10-fold cross-validation conducted on the BT session, comparing NMA and FBCSP. Apart from Pipeline 0, every pipeline utilizing NMA demonstrates notable improvements across different subjects, consistently achieving higher accuracy compared with the FBCSP. The parameters learned during the BT session were subsequently used to evaluate performance on the BE session, which served as a test set. Table 2 presents the results obtained from the BE session on the Graz Dataset 2b, with the trained models assessing generalization capabilities in a new session. A new model is added for comparison, a ShallowConvNet (SCN) architecture [33]. SCN consists of two convolutional layers (temporal, then spatial), a squaring nonlinearity ($f\left(x\right)=x^{2}$), an average pooling layer, and log nonlinearity (f(x)=log(x)). The SCN architecture was specifically designed for oscillatory signal classification (by extracting features related to log-band power). The accuracy results on the BE session show that the pipelines introduced in this paper outperform both FBCSP and SCN in the majority of subjects, demonstrating superior classification accuracy in distinguishing between left and right hand movements. This dataset effectively serves as a testbed, illustrating that even with only two classes, the NMA approach can successfully identify intervals that enhance feature separability. The following section extends this approach to a multi-class scenario, where NMA is used to isolate intervals that improve the separability of specific classes that are otherwise poorly distinguishable.

### 3.2. Tests on Graz Dataset 2a

This subsection provides a brief overview of the Graz Dataset 2a and compares the performance improvements introduced by NMA in a multi-class problem to other approaches, such as FBCSP and SCN [33], both of which have demonstrated effectiveness on this dataset. Additionally, this section demonstrates how features extracted from one subject can be effectively leveraged to improve classification performance in another subject.

#### 3.2.1. Graz Dataset 2a Description

The Graz Dataset 2a [43] for the BCI Competition IV is specifically designed to systematically study EEG responses associated with various motor imagery tasks, thereby facilitating the analysis of brain activity patterns. This dataset is particularly valuable due to its complexity, involving four distinct motor imagery classes.

The dataset includes EEG data from nine subjects participating in a cue-based BCI paradigm involving four motor imagery tasks: imagining the movement of the left hand (class 1), right hand (class 2), both feet (class 3), and tongue (class 4). During the experiment, subjects sat in front of a computer screen. Each subject completed two sessions, named A Training (AT) and A Evaluation (AE), recorded on different days. Each session includes recordings from three EOG channels and twenty-two EEG channels. However, only the EEG channels were considered. The registered session consists of six runs separated by short breaks. Each run included 48 trials (12 for each class), resulting in a total of 288 trials per session.

**Figure 3:**
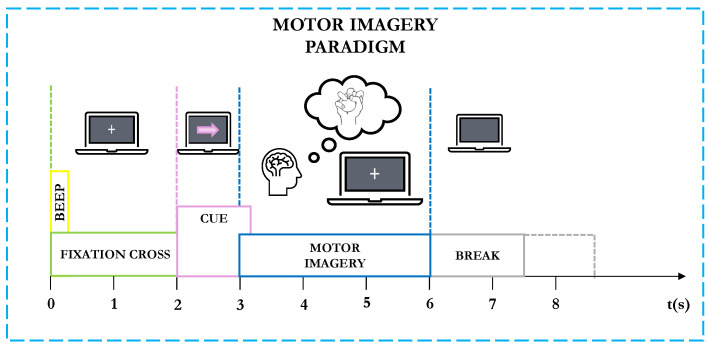
Graz Dataset 2a MI Task: timing scheme of the paradigm: subjects sat in front of a computer screen. After *t* = 2 s, a cue in the form of an arrow (left, right, down, or up) appeared for *t* = 1.25 s, indicating the MI task to perform. Subjects were instructed to continue the motor imagery task until the fixation cross disappeared at *t* = 6 s, followed by a short break with a black screen.

At the start of each trial (t=0 s), a fixation cross appeared on the black screen, accompanied by a short acoustic warning tone. After two seconds (t=2 s), a cue in the form of an arrow (left, right, down, or up) appeared for 1.25 s, indicating the motor imagery task to perform. Subjects were instructed to continue the MI task until the fixation cross disappeared at t=6 s, followed by a short break with a black screen (see Figure 3 for a detailed depiction of the task recording). The dataset is open and is freely available at www.bbci.de/competition/iv/ (accessed on 02 February 2024).

#### 3.2.2. Enhancing Class Separation via NMA

**Figure 4:**
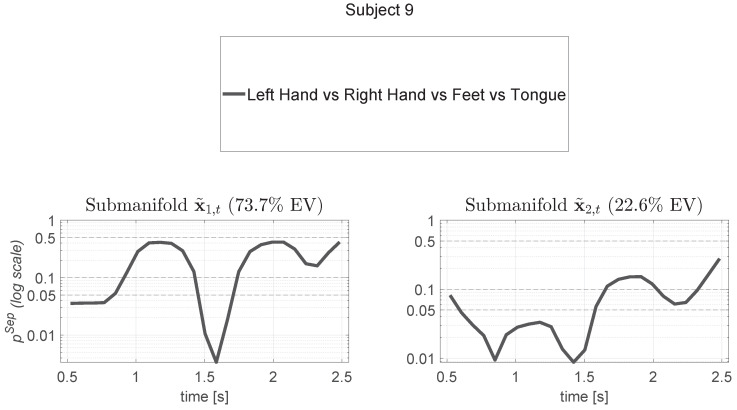
Results for Subject 9 showing the computation of the separability measure of the manifold trajectories among all classes. We present the probability $p^{Sep}\left(t\right)$ of trajectory separation under the null hypothesis of no effect among classes. Above each plot, the percentage of explained variance (EV) is reported for each manifold direction, with two manifold directions accounting together for over 95% of the variance. A one-way ANOVA [63] was performed at each time step among the values $c_{k}\left(t\right)$, yielding a *p*-value that quantifies the separability of the classes over time. The closer this *p*-value is to zero, the more separable the classes are. Based on these values, the time *s* of maximum separability among classes ($\arg\min_{t\in T}p^{Sep}\left(t\right)$) can be identified.

NMA allows for the detection of features that are more discriminative concerning motor imagery tasks. By applying the NMA procedure to each subject, subject-specific intervals such as *T*, $T_{s}$, $T_{s_{ij}}$, and $T_{s_{ij}}^{0}$ can be identified, enabling the refinement of features for the classifier. A series of figures is provided to clearly illustrate the method. In Figure 4, the results for a sample subject (Subject 9) are reported, depicting the computation of the separability measure of the trajectories among all classes. For this subject, two directions explain more than 95% of the variance. Panels a and b in Figure 4 display the separability measure for all classes across two directions. Lower values indicate greater separation, allowing us to identify optimal times (e.g., *T* and $T_{s}$; see Section 2) for centering the time series analysis.

**Figure 5:**
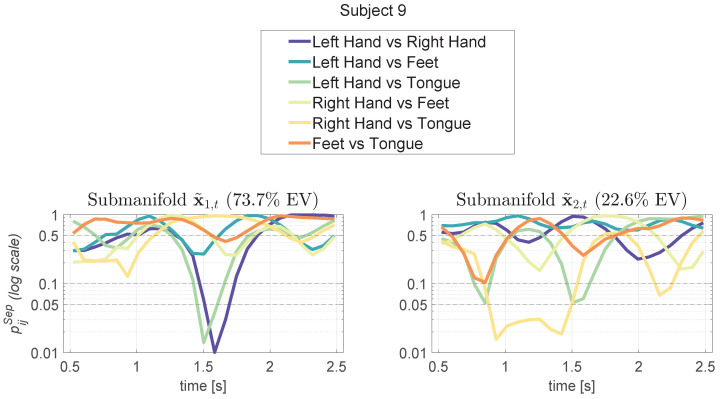
Results for Subject 9, showing the computation of the separability measure of the manifold trajectories one class versus the other, following the one-way ANOVA test (see Figure 4). A post hoc Tukey test was used to obtain the separability measure $p_{ij}^{Sep}\left(t\right)$ between two distinct classes. By analyzing the trend of these measures, the minimum values where the classes are most separated can be identified. Based on these values, the time $s_{ij}$ of maximum separability between class *i* and class *j* ($\arg\min_{t\in T\;\mathrm{and}\;p^{Sep}\left(t\right)<0.05}p_{ij}^{Sep}\left(t\right)$) can be determined.

Similarly, Figure 5 illustrates the separability measure of the trajectories between pairs of specific classes for the same subject. This is again shown for two directions, where lower values signify greater separation, enabling the identification of times (e.g., $T_{s_{ij}}$, $T_{s_{ij}}^{0}$, see Section 2) for focusing the time series analysis.

**Figure 6:**
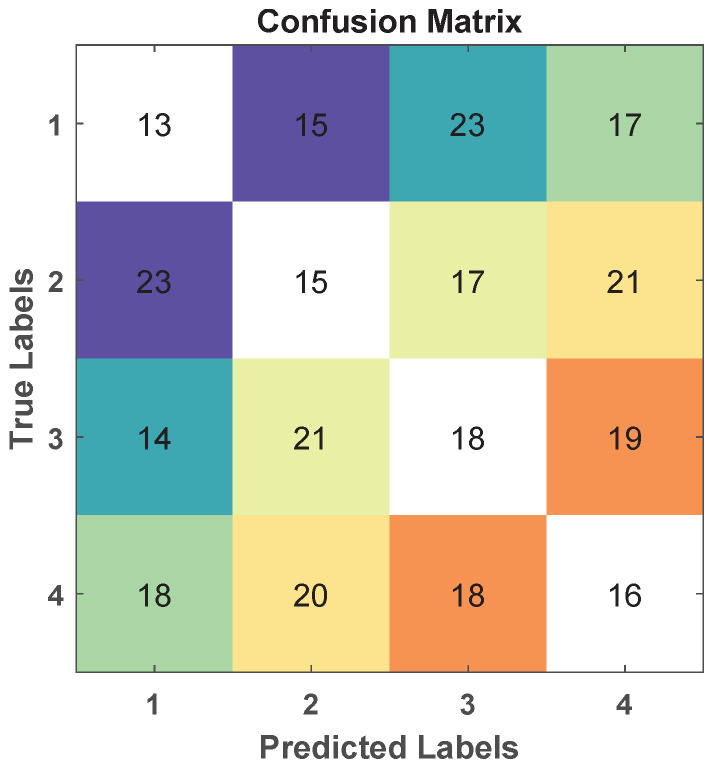
Confusion Matrix $A_{ij}$ obtained with FBCSP for a sample Subject 9 for the four classes: (1) left hand, (2) right hand, (3) feet, and (4) tongue. Each row represents the true instances in class *i*, while each column represents the instances in the predicted class *j*. Thus, the diagonal elements $A_{ii}$ represent correctly predicted instances, while the off-diagonal elements correspond to misclassifications. In the figure, each distinct color highlights the reciprocal misclassification between pairs of classes, visually illustrating the degree of confusion in classification results. Consequently, $\arg\max_{i,i}\left(A_{ij}+A_{ji}\right)$ gives the value of the worst class pair, in this case, 2 vs. 4 (right hand vs. tongue). Consequently, it is possible to select the interval $T_{s_{ij}}$ based on the separability between these two classes detected by NMA.

From the classification phase, confusion matrices for the four classes can be constructed for each subject using both FBCSP and Pipeline 0. Figure 6 shows the confusion matrix obtained with FBCSP for a sample Subject 9. These confusion matrices are utilized to detect the intervals $T_{s_{ij}}$ and $T_{s_{ij}}^{o}$, respectively, based on the minimum separability measure between two classes detected by NMA.

**Figure 7:**
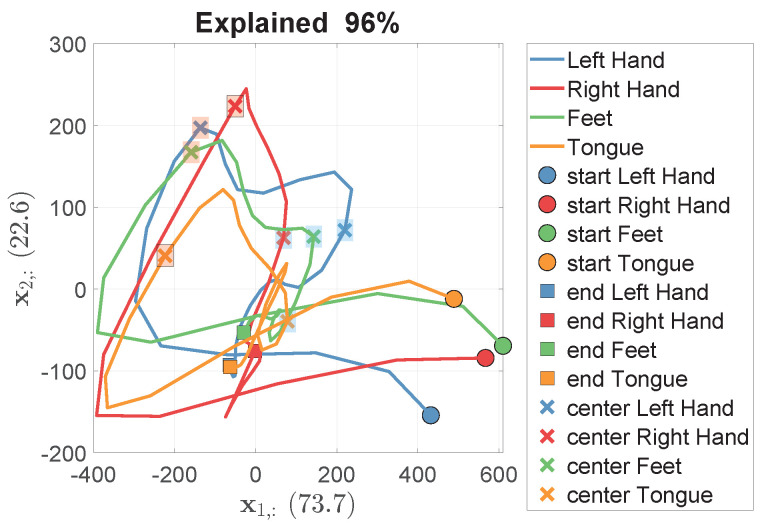
Two−dimensional trajectories of the coefficients ${\bar{c}}_{k}^{1}\left(t\right)$ against ${\bar{c}}_{k}^{2}\left(t\right)$ for the manifold directions of Subject 9, averaged for each class, where *k* corresponds to one of the four motor imagery classes: left hand, right hand, feet, and tongue. These plots highlight the points of maximum separation among the classes, indicated by crosses in blue rectangles, and specifically between right hand and tongue, indicated by crosses in red rectangles (note that black borders are drawn around the right hand and tongue points).

**Figure 8:**
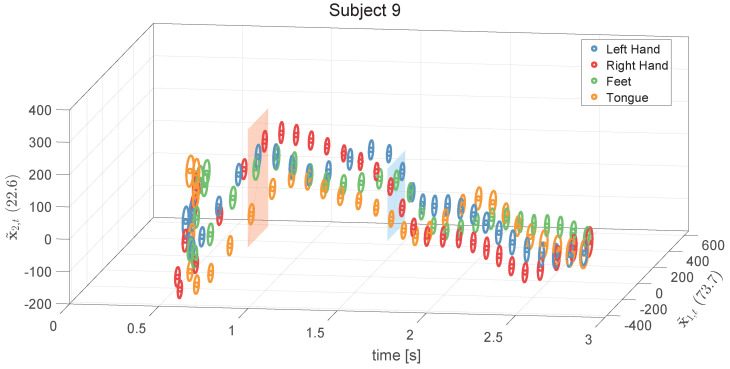
Two−dimensional ellipsoids for manifold directions of Subject 9, showing coefficients ${\bar{c}}_{k}^{1}\left(t\right)$ and ${\bar{c}}_{k}^{2}\left(t\right)$ against time, where *k* corresponds to one of the four motor imagery classes: left hand, right hand, feet, and tongue. The dimensions of the ellipsoids are based on the standard deviation for each manifold sub-dimension. This illustration further highlights the points of maximum separation among the classes, and specifically between the right hand and tongue classes.

To further clarify the analysis for Subject 9, 2D trajectories of the coefficients ${\bar{c}}_{k}^{1}\left(t\right)$ against ${\bar{c}}_{k}^{2}\left(t\right)$, averaged for each class, are plotted (see Figure 7), where *k* corresponds to one of the four motor imagery classes: left hand, right hand, feet, and tongue. These plots highlight the points of maximum separation among the classes (crosses in blue rectangles) and between right hand and tongue (crosses in red rectangles). The same trajectories are also depicted as 2D ellipsoids (see Figure 8), illustrating ${\bar{c}}_{k}^{1}\left(t\right)$ and ${\bar{c}}_{k}^{2}\left(t\right)$ against time, with the ellipsoid dimensions based on the standard deviation for each manifold sub-dimension. This further illustrates the points of maximum separation among classes, specifically between right hand and tongue.

Table 3 shows the results from the AT session of the Graz Dataset, performing 10-fold cross-validation. Each pipeline involving NMA demonstrates improvements in different subjects. Apart from Pipeline 0, each pipeline shows improvements in accuracy compared with the FBCSP winner of the BCI competition. Moreover, by choosing the most successful pipeline for each subject, it is evident that the presented method brings significant improvements over the state-of-the-art algorithm.

The same comparison was conducted on the AE session, utilizing the model trained on the AT session to evaluate its generalization capabilities in a new session. This aligns with the spirit of the BCI competition, where the AE session served as the test set for comparing methods, and the AT session was used for system tuning. As shown in Table 4, the accuracy results are lower in the AE session compared with the AT session, as expected. However, the presented method consistently defines pipelines that outperform the competition winner and demonstrate better accuracy than the ones of FBCSP and SCN. Interestingly, in the case of four-class classification, the performance gap between NMA-based pipelines and other approaches appears to widen. This suggests that as the number of classes increases, our separability properties allow the NMA-based approach to construct manifolds that more effectively distinguish among different conditions, leading to improved discrimination. By selecting the best pipeline for each subject, the proposed method achieves superior accuracy compared with the FBCSP and SCN approaches, which have been used as benchmarks for SMR classification.

#### 3.2.3. Cross-Subjects Manifold Sharing

Until now, each result was obtained by analyzing the manifolds of specific subjects and attempting to improve classification by enhancing specific features whenever NMA detected them. However, this section shows that this analysis can be extended further. By leveraging the capabilities of certain subjects who excel in class discrimination, it is possible to augment the performance of subjects with poorer classification abilities. Specifically, this approach imports results of MNA from high-performing subjects and projects them onto other subjects. This approach combines previous BCI systems (explained in the previous subsection) with new features detected with the aid of other successful subjects.

**Figure 9:**
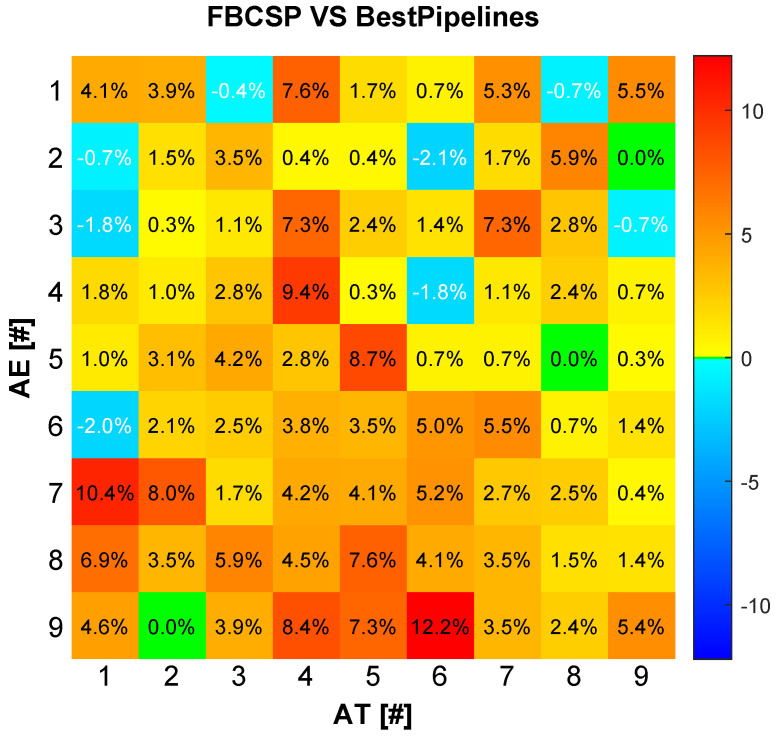
The figure illustrates the results of the cross−subject NMA approach. The confusion matrix presents the classification performance when subject *i* uses NMA insights from subject *j*. Each matrix element $A_{ij}$ indicates the percentage improvement in accuracy compared with the FBCSP benchmark. Diagonal elements represent pipelines using only NMA from the same subject, while off-diagonal elements show results using pipelines augmented with the best features obtained through NMA analysis from other successful subjects.

Figure 9 illustrates the results of this hybridization approach. This combined confusion matrix shown presents the classification performance when subject *i* uses an NMA insight *j* from another subject. Each matrix element $A_{ij}$ indicates the percentage improvement in accuracy compared with the FBCSP and SCN benchmarks. The diagonal elements represent pipelines using only NMA from the same subject, while the off-diagonal elements show results using pipelines augmented with the best features obtained through NMA analysis from other successful subjects.

This figure demonstrates that augmenting features by NMA from other subjects significantly enhances the BCI system’s classification capabilities. This approach highlights the potential of cross-subject NMA information sharing in improving overall system performance, showcasing how knowledge transfer among subjects can lead to better generalization and more robust BCI systems. Table 5 shows the accuracies on the AE session when using the best NMA result per subject (computed in the previous subsection) and the best NMA result across subjects. The results demonstrate that NMA information shared across subjects can further improve classification performance.

**Figure 10:**
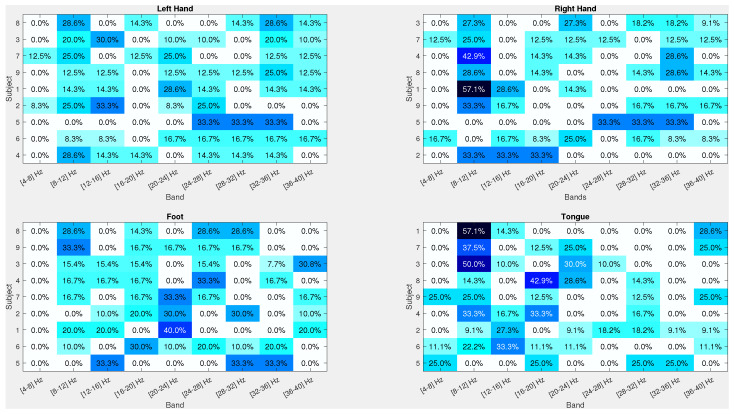
Statistics of the successful frequency bands for each subject, sorted and obtained from the CSP projection matrix of the first selected feature per subject. The figure presents the distribution of the most effective frequency bands that contribute to the classification accuracy across different subjects. Each rectangle represents the frequency band that was most frequently selected for optimal feature extraction, highlighting the variability and commonality of effective frequency ranges among subjects. This analysis underscores the significance of individual–specific frequency bands in enhancing the performance of motor imagery tasks in BCI systems. The detailed examination of these bands provides valuable insights into the neural oscillatory patterns that are critical for accurate classification. For further details, refer to the main text.

**Figure 11:**
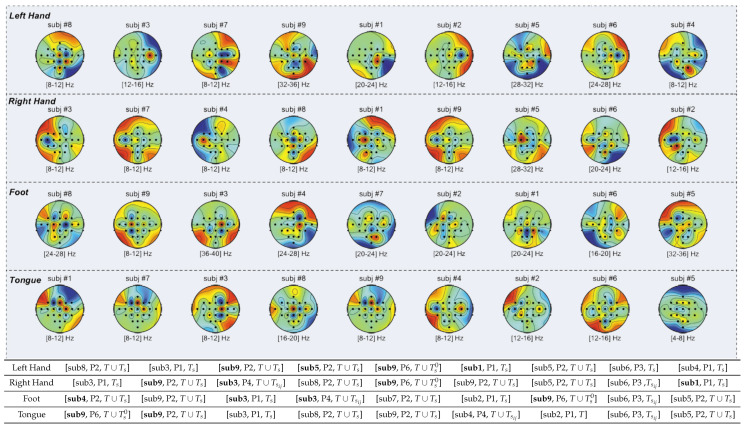
Topographic maps obtained from the CSP projection matrix for the first selected feature per subject. These maps illustrate the best feature per class, ordered by classification accuracy per class. The topographic maps highlight characteristic spatial patterns for different motor imagery tasks: contralateral patterns for left and right hand movements, top–central activation for foot movements, and parietofrontal activation for tongue movements. In the accompanying table, three values are indicated: the subject from which the feature is derived (with features borrowed from another subject highlighted in bold), the Pipeline $P_{l}$ from which the feature is extracted, and the reference interval to which the feature corresponds (see Section 2.6). It is notable that several features are borrowed from other subjects, and certain subjects contribute more frequently to these feature–lending scenarios. This suggests that features from high–performing subjects can be effectively used to prototype efficient BCI systems.

Finally, to provide insight into the learned features, the frequency bands (see Figure 10) and topographic maps (Figure 11) are presented, both obtained from the CSP projection matrix of the first selected features. These figures illustrate the characteristic patterns for different motor imagery tasks: a contralateral pattern for left hand and right hand, top-central activation for feet [8,9], and parietofrontal activation for tongue [44]. Notably, several features are “borrowed” from other subjects, demonstrating that cross-subject features can significantly enhance accuracy. This cross-subject feature borrowing is particularly beneficial in improving the robustness and generalization of the BCI system, making it more adaptable to various users. The integration of these features across subjects highlights the potential of NMA to uncover critical patterns that are not only subject-specific but also generalizable across different individuals, further strengthening the classification performance and reliability of EEG-based BCI systems.

## 4. Conclusions

The presented study demonstrates the significant potential of integrating NMA with traditional EEG-based MI-BCI systems to enhance feature extraction and classification accuracy. By identifying specific time intervals that capture class-specific and subject-specific characteristics, the presented approach has demonstrated the ability to enhance the performance of MI-BCI systems, particularly for challenging subjects. This method not only refines the features for individual subjects but also leverages cross-subject information to further boost classification accuracy. The primary objective of this paper was to develop a method specifically designed for classifying the oscillatory components of sensorimotor rhythms during MI tasks. The Graz Datasets 2a and 2b, widely recognized benchmarks in this field, were utilized to validate the proposed approach. This work builds on the most efficient existing methods tailored for MI tasks (such as the FBCSP algorithm, the winner of the BCI competition, and SCN, specifically designed for MI tasks). By introducing NMA-based preprocessing to create novel MI-BCI pipelines, the discriminability of trials is significantly improved, as features become more separable with respect to distinct motor imagery classes. The presented results demonstrate that incorporating NMA in the preprocessing stage enhances the performance of established algorithms. As detailed in Section 3, the results underscore the robustness and adaptability of the presented approach, paving the way for more reliable and efficient MI-BCI systems.

Future research directions could involve incorporating successful neural network approaches, such as SCN, which has been shown to be effective for SMR classification, by completely replacing the CSP modules within the pipeline. While DL techniques typically automate feature extraction and selection, they could potentially benefit from a preprocessing phase that begins with manifold analysis. Furthermore, an intriguing possibility would be to develop a conditional VAE [66]—a deep VAE model conditioned on the separability properties in NMA outlined in our Methods section. This approach could further optimize the feature selection process, contributing to more refined and user-friendly BCI applications.

## Figures and Tables

**Table 1:** Accuracy results from the BT sessions of the Graz 2b Dataset, performing 10-fold cross-validation. Each pipeline involving NMA demonstrates improvements in different subjects. Apart from Pipeline 0, each pipeline shows increased accuracy compared with the FBCSP. Values for pipelines that outperform FBCSP are in bold. The best pipeline per subject is highlighted in green.

BT Session					
**Accuracy [%]**					
**Subject [#]**	**FBCSP**	**Pipeline 0**	**Pipeline 1**	**Pipeline 2**	**Best Pipelines**
1	71.6	65.8	**74.0**	**73.0**	**74.0**
2	55.2	**61.5**	**58.0**	**60.3**	**60.3**
3	61.3	53.3	58.8	**62.0**	**62.0**
4	93.4	89.3	92.4	**94.0**	**94.0**
5	83.3	81.4	**84.0**	**83.8**	**84.0**
6	72.2	68.0	**73.3**	**74.3**	**74.3**
7	73.1	**73.8**	**78.0**	**76.5**	**78.0**
8	65.1	**68.2**	**68.4**	**66.8**	**68.4**
9	70.9	69.0	**72.8**	71.0	**72.8**
**mean ± SE**	71.8 ± 3.8	70.0 ± 3.5	**73.3 ± 3.7**	**73.5 ± 3.5**	**74.2 ± 3.5**

**Table 2:** Results from the BE session of Graz Dataset 2b, using models trained on the BT session to assess generalization capabilities in a new session, are presented. Accuracy results higher than those of FBCSP and SCN are shown in bold. The best approach for each subject is highlighted in red.

BE Session						
**Accuracy [%]**						
**Subject [#]**	**FBCSP**	**SCN**	**Pipeline 0**	**Pipeline 1**	**Pipeline 2**	**Best Pipelines**
1	66.3	76.2	64.1	65.0	65.3	65.3
2	56.1	51.0	55.4	**58.6**	**57.9**	**58.6**
3	51.3	53.4	**55.3**	**59.1**	**58.8**	**59.1**
4	94.4	95.7	95.9	**96.6**	**95.9**	**96.6**
5	87.2	87.2	83.1	**88.8**	86.3	**88.8**
6	76.3	77.6	65.6	77.2	**78.1**	**78.1**
7	75.6	76.3	**86.3**	**78.8**	74.7	**86.3**
8	86.3	75.6	86.3	**89.4**	**87.5**	**89.4**
9	82.8	86.3	77.5	82.8	80.6	82.8
**mean ± SE**	75.1 ± 4.9	76.8 ± 5.1	74.4 ± 4.9	**77.3 ± 4.4**	76.1 ± 4.4	**78.3 ± 4.7**

**Table 3:** Accuracy results from the AT session of the Graz Dataset 2a, performing 10-fold cross-validation. Each pipeline involving NMA demonstrates improvements in different subjects. Apart from Pipeline 0, each pipeline shows increased accuracy compared with the FBCSP winner of the BCI competition (considered as a benchmark). Values for pipelines that outperform FBCSP are in bold. The best pipeline per subject is highlighted in green. Notice that apart from Pipeline 0, each pipeline achieves higher average accuracy across subjects compared with FBCSP.

AT Session									
**Accuracy [%]**									
**Subject [#]**	**FBCSP**	**Pipeline 0**	**Pipeline 1**	**Pipeline 2**	**Pipeline 3**	**Pipeline 4**	**Pipeline 5**	**Pipeline 6**	**Best Pipelines**
1	78.1	66.3	77.4	76.0	**80.9**	**79.5**	75.3	**79.9**	**80.9**
2	46.5	45.1	**49.7**	46.2	**49.3**	**50.7**	**49.3**	**53.1**	**53.1**
3	81.9	75.0	**84.7**	**83.7**	79.5	**86.8**	81.9	**87.2**	**87.2**
4	49.3	49.0	**54.5**	**51.4**	**53.5**	**52.8**	**52.1**	**54.5**	**54.5**
5	58.0	56.3	55.6	**65.5**	54.9	**60.1**	53.8	**59.0**	**65.5**
6	50.0	**52.4**	**58.0**	**56.3**	**52.8**	**53.5**	**55.6**	**52.8**	**58.0**
7	78.8	77.8	**80.6**	**84.4**	**79.2**	76.7	76.7	**79.5**	**84.4**
8	85.4	84.7	**88.9**	83.0	83.0	**86.8**	83.7	85.1	**88.9**
9	83.3	80.2	83.0	79.5	78.8	**85.1**	**86.5**	83.7	**86.5**
**mean ± SE**	67.9 ± 5.5	65.2 ± 5.0	**70.3 ± 5.2**	**69.5 ± 5.0**	**68.0 ± 4.9**	**70.2 ± 5.2**	**68.3 ± 5.1**	**70.5 ± 5.0**	**73.2 ± 5.1**

**Table 4:** Results on the AE session of Graz Dataset 2a, using the models trained on the AT session to assess generalization capabilities in a new session. Accuracy results higher than those of FBCSP and SCN are shown in bold. The best approach for each subject is highlighted in red.

AE Session										
**Accuracy [%]**										
**Subject [#]**	**FBCSP**	**SCN**	**Pipeline 0**	**Pipeline 1**	**Pipeline 2**	**Pipeline 3**	**Pipeline 4**	**Pipeline 5**	**Pipeline 6**	**Best Pipelines**
1	67.8	71.4	57.3	67.7	66.7	67.7	71.2	71.2	**71.9**	**71.9**
2	47.5	39.2	46.9	**49.0**	**48.3**	45.1	**49.0**	44.1	46.2	**49.0**
3	83.3	82.5	77.1	**84.4**	82.3	83.7	83.7	82.6	81.3	**84.4**
4	53.5	58.7	53.1	57.6	58.3	55.6	**63.2**	54.5	57.6	**63.2**
5	32.4	44.3	38.2	36.8	43.1	35.8	39.2	36.5	38.5	43.1
6	42.6	46.9	41.3	42.7	43.8	**47.9**	42.4	42.0	43.4	**47.9**
7	80.6	76.9	74.7	78.1	**83.3**	78.1	78.8	78.1	78.1	**83.3**
8	82.2	74.8	77.1	79.2	80.6	81.3	80.9	80.9	80.2	80.9
9	71.7	75.9	70.1	71.5	**77.1**	71.5	73.3	**77.1**	**77.1**	**77.1**
**mean ± SE**	62.4 ± 6.3	63.4 ± 1.9	59.5 ± 5.2	63.0 ± 5.7	**64.8 ± 5.6**	63.0 ± 5.8	**64.6 ± 5.7**	63.0 ± 6.2	**63.8 ± 5.8**	**66.7 ± 5.5**

**Table 5:** Comparison of accuracies on the AE session of Graz Dataset 2a between the FBCSP and SCN benchmarks, the best NMA result per subject, and the best NMA result across subjects. The values clearly demonstrate that sharing NMA information across subjects can further improve classification performance. The highest accuracy for each subject is highlighted in bold.

AE Session				
	**FBCSP**	**SCN**	**NMA per Subject**	**NMA Cross Subjects**
**Subject [#]**	**Accuracy [%]**			
1	67.8	71.4	**71.9**	**72.6**
2	47.5	39.2	**49.0**	**51.7**
3	83.3	82.5	**84.4**	**84.4**
4	53.5	58.7	**63.2**	**64.2**
5	32.4	**44.3**	43.1	43.1
6	42.6	46.9	**47.9**	**48.6**
7	80.6	76.9	**83.3**	**84.4**
8	**82.2**	74.8	80.9	81.3
9	71.7	75.9	**77.1**	**78.5**
**mean ± SE**	62.4 ± 6.3	63.4 ± 5.5	**66.7 ± 5.5**	**67.6 ± 5.4**

## Data Availability

Data are available at: www.bbci.de/competition/iv/.
